# C-di-AMP Is a Second Messenger in *Corynebacterium glutamicum* That Regulates Expression of a Cell Wall-Related Peptidase via a Riboswitch

**DOI:** 10.3390/microorganisms11020296

**Published:** 2023-01-23

**Authors:** Sebastian J. Reich, Oliver Goldbeck, Tsenguunmaa Lkhaasuren, Dominik Weixler, Tamara Weiß, Bernhard J. Eikmanns

**Affiliations:** 1 Institute of Microbiology and Biotechnology, Ulm University, 89081 Ulm, Germany; 2 Institute of Biochemistry, Department of Chemistry, University of Cologne, 50674 Cologne, Germany

**Keywords:** *Corynebacterium glutamicum*, c-di-AMP, second messenger, diadenylate cyclase, phosphodiesterase, riboswitch, cell wall, regulation

## Abstract

Cyclic di-adenosine monophosphate (c-di-AMP) is a bacterial second messenger discovered in *Bacillus subtilis* and involved in potassium homeostasis, cell wall maintenance and/or DNA stress response. As the role of c-di-AMP has been mostly studied in Firmicutes, we sought to increase the understanding of its role in Actinobacteria, namely in *Corynebacterium glutamicum*. This organism is a well-known industrial production host and a model organism for pathogens, such as *C. diphtheriae* or *Mycobacterium tuberculosis*. Here, we identify and analyze the minimal set of two *C. glutamicum* enzymes, the diadenylate cyclase DisA and the phosphodiesterase PdeA, responsible for c-di-AMP metabolism. DisA synthesizes c-di-AMP from two molecules of ATP, whereas PdeA degrades c-di-AMP, as well as the linear degradation intermediate phosphoadenylyl-(3′→5′)-adenosine (pApA) to two molecules of AMP. Here, we show that a *ydaO/kimA*-type c-di-AMP-dependent riboswitch controls the expression of the strictly regulated cell wall peptidase gene *nlpC* in *C. glutamicum*. In contrast to previously described members of the *ydaO/kimA*-type riboswitches, our results suggest that the *C. glutamicum nlpC* riboswitch likely affects the translation instead of the transcription of its downstream gene. Although strongly regulated by different mechanisms, we show that the absence of *nlpC*, the first known regulatory target of c-di-AMP in *C. glutamicum*, is not detrimental for this organism under the tested conditions.

## 1. Introduction

Bacteria use several different nucleotide-derived molecules as second messengers for the control of important cellular functions [1]. Of these, 3′,5′-cyclic diadenosine monophosphate (c-di-AMP) has an outstanding role as it is the only known nucleotide second messenger, which seems to be essential for bacterial survival, at least under most standard laboratory conditions [2,3,4,5]. In Firmicutes, such as *Bacillus subtilis*, *Staphylococcus aureus* and *Listeria monocytogenes*, where c-di-AMP has mostly been studied, this molecule fulfils a major role in the control of ion homeostasis, but its implication in cell wall synthesis, DNA integrity and sporulation has also been shown [5,6,7,8,9,10]. Furthermore, c-di-AMP is also involved in virulence and host interactions in pathogens, such as *Listeria* and *Chlamydia* [11,12,13]. C-di-AMP has been first detected during the structural investigation of the DNA integrity scanning protein A (DisA) of *Thermotoga maritima* and *B. subtilis* [14]. The homo-octameric enzymes synthesize c-di-AMP by joining two adenosine triphosphate (ATP) molecules via phosphodiester linkages and have been described as migrating along DNA, where they interact with stalled replication forks and Holliday junctions in *B. subtilis* [14,15]. Up to now, different types of such diadenylate cyclases (DACs) have been identified, which all contain the so-called ‘DAC domain’ [16]. As DisA, the two other important types of DACs were also discovered in *B. subtilis*: The membrane-bound CdaA, which seems to be linked to potassium/ion homeostasis, and the small soluble CdaS involved in sporulation processes [4,17]. In contrast to *B. subtilis*, other organisms often possess only one DAC. For example, Firmicutes, such as *L. monocytogenes* or *S. aureus*, contain only a CdaA homolog, whereas Actinobacteria seem to be limited to DisA homologs [16,18]. However, DAC-encoding genes (and thus c-di-AMP signaling mechanisms in general) are widely spread, mostly represented in Gram-positive bacteria, but they are also found in Gram-negatives, such as Cyanobacteria and Chlamydiae, and even in the kingdom of archaea [19].

In contrast to DACs, the phosphodiesterases (PDEs) involved in c-di-AMP degradation instead show more structural diversity [16]. Here, two-step degradation via the linear intermediate 5′-phosphoadenylyl-(3′→5′)-adenosine (pApA) and one-step mechanisms via direct hydrolysis of two molecules of AMP have been described [20,21]. In contrast to DAC activity, c-di-AMP cleavage cannot be attributed to one particular domain, but instead it is mediated by various completely different structures [16]. Although several enzymes share similarities in domain architecture, such as the well-characterized DhhP-type PDEs first described in *Borrelia burgdorferi* [22,23], others appear significantly different, such as the recently discovered AtaC from *Streptomyces venezuelae*, which does not contain any of the domains described to be involved in c-di-AMP hydrolysis previously [24].

The mechanisms by which c-di-AMP regulates cellular processes include protein interaction, e.g., as ligands of transcriptional regulators, such as BusR of *Lactococcus lactis*, or with regulatory domains of enzymes, e.g., the potassium transporter KtrA in *S. aureus* [7,25]. In addition, it was also described to act as an effector molecule of c-di-AMP-specific riboswitches [26]. The first described member of these c-di-AMP riboswitches is located in the 5′ untranslated region (UTR) of the *ydaO* gene in *B. subtilis*, which encodes for the potassium importer KimA [4,26]. Upon the binding of c-di-AMP, the formation of a terminator stem loop occurs, which then results in premature transcription termination [26,27]. Thus, c-di-AMP riboswitches are considered to act as so-called ‘off’-switches [26]. Although the upstream of a potassium transporter gene were discovered first, many of these riboswitches have been predicted to be located in front of a variety of genes across Gram-positive bacteria. Whereas in Bacillales and Clostridia over 50% of these genes seem to encode various ions, osmoprotectants or amino acid transporters, almost all of the *ydaO*-preceded genes in Actinobacteria seem to be cell wall related [26]. This has also been further investigated in *Streptomyces coelicolor*, where a c-di-AMP-dependent *ydaO*-type riboswitch in front of the resuscitation-promoting factor gene *rpfA* contributes to a complex multilevel regulatory mechanism, also involving other nucleotides, such as cAMP and ppGpp [28]. However, in contrast to Firmicutes, profound knowledge about the role of c-di-AMP in Actinobacteria is still limited, i.e., to several reports about *Streptococcus* and *Mycobacterium* species, e.g., [24,28,29,30]. Whereas in Streptococci, c-di-AMP seems to play a role in cell wall maintenance and in ion homeostasis, reports about Mycobacteria suggest an implication in ensuring DNA integrity [24,28,31]. The Gram-positive *Corynebacterium glutamicum* is a non-pathogenic Actinobacterium, known for its wide usage in industrial biotechnology, i.e., amino acid production (for reviews, see [32,33]). In this context, not only have its metabolic pathways been extensively studied but its specific and global regulatory pathways are also well investigated (see reviews [34,35,36,37]). Thus, it represents a well-characterized model organism for its close relatives, such as the human pathogenic *Corynebacterium diphtheriae* and *Mycobacterium tuberculosis*. In their study, Nelson et al. [26] predicted two putative *ydaO*-type riboswitches in the genome of *C. glutamicum*, one of them located upstream of the *rpf1* gene, which is similar as for *rpfA* in *S. coelicolor* and the second upstream of the *cg2402*/*nlpC* gene. This gene encodes for one of four putative NlpC/P60-type cell wall peptidases in *C. glutamicum* [38,39,40] and is the first gene of an operon comprising, among others, a second *nlpC*-type gene (*cg2401*). The operon seems to be strongly regulated, i.e., by the global master regulator GlxR, the two-component system MtrAB and the three-component system EsrISR [39,41,42] and thus represents an interesting topic for investigation. In our study, we initially characterized c-di-AMP metabolism in *C. glutamicum* and describe a non-canonical c-di-AMP riboswitch as the first regulatory target of c-di-AMP in this organism.

## 2. Materials and Methods

### 2.1. Bacterial Strains, Plasmids and Culture Conditions

The bacterial strains and plasmids used in this study are listed in Table 1. Pre-cultures of *Escherichia coli* and *C. glutamicum* strains were grown in 50 mL 2xTY complex medium (16 g tryptone, 10 g yeast extract and 5 g sodium chloride per liter; [43]) in baffled Erlenmeyer flasks at 130 rpm and 37 or 30 °C, respectively. Main cultures of *C. glutamicum* were grown in modified CGXII medium [44] with 20 g (NH_4_)_2_SO_4_ and 10 g glucose per liter at pH 7 and 30 °C. When appropriate, kanamycin (50 µg mL^−1^), chloramphenicol (12.5 µg mL^−1^ for *C. glutamicum*, 25 µg mL^−1^ for *E. coli*), isopropyl β-D-1-thiogalactopyranoside (IPTG, 0.1 mM for *C. glutamicum*, 1 mM for *E. coli*), anhydrotetracycline (250 ng mL^−1^) or arabinose (1% *w*/*v*) was added.

### 2.2. Construction of Plasmids and Recombinant Strains

Plasmid construction was performed using molecular cloning methods [43] and DNA assembly described by Gibson et al. [54]. Restriction enzymes, alkaline phosphatase and T4 DNA ligase were purchased from Thermo Scientific (Waltham, MA, USA); Q5 high fidelity polymerase was purchased from NEB (Ipswich, MA, USA), RedMastermix from Genaxxon bioscience GmbH (Ulm, Germany). All enzymes were used according to the manufacturer’s protocol. Deoxynucleotide triphosphates were purchased from BioBudget (Krefeld, Germany) and oligonucleotides from biomers.net GmbH (Ulm, Germany). Oligonucleotide sequences are listed in Table S1. PCR was performed using a C100 thermocycler (Bio-Rad Laboratories, Munich, Germany). PCR conditions were chosen according to primer composition and fragment length. PCR products were separated in agarose gels and purified using the Nucleo-Spin DNA extraction kit (Macherey-Nagel, Düren, Germany). Detailed plasmid construction descriptions are included in the supplementary material. For transformation of *E. coli* DH5α, standard methods were applied [55]. Transformation of *C. glutamicum* was performed by electroporation, as described before [56,57]. Chromosomal deletions in *C. glutamicum* were performed using the CRISPR-Cpf1 method based on Jiang et al. [52]. Recombinant strains were selected on 2xTY agar containing the respective antibiotic.

### 2.3. Recombinant Protein Production and Purification

Recombinant C-terminal Strep-tagged DisA and PdeA were produced using *E. coli* BL21 carrying the respective expression plasmid (Table 1). Cells were grown in 100 mL terrific broth containing 50 µg kanamycin mL^−1^ at 37 °C and 130 rpm. Induction was done with 1 mM IPTG at an optical density at 600 nm (OD_600_) of 3–4. Cultivation was continued overnight and cells were then harvested by centrifugation. The pellet was then washed twice with 0.9% NaCl and resuspended in 10 mL of the appropriate extraction buffer containing 50 mM Tris at neutral pH, 150 mM NaCl and EDTA-free protease inhibitor (Roche, Mannheim, Germany). The cells were disrupted using a Sonifier 250 (Branson, Danbury, CT, USA) by five sonication cycles of 30 s (duty cycle: 25%, output control: 4) with cooling on ice in between. The lysate was cleared by centrifugation at 4 °C, and the proteins were purified, according to the respective manufacturer’s manual. The Strep-tagged proteins were then purified using 2 mL gravity flow columns (Strep-Tactin Sepharose, IBA, Göttingen, Germany), according to the manufacturer’s manual.

### 2.4. Diadenylate Cyclase and Phosphodiesterase Assays

To determine DAC activity of DisA or PDE activity of PdeA, assays were performed based on the methods described by Manikandan et al. [30]. In particular, 10 µM of DisA-Strep or 0.2 µM PdeA-Strep was incubated in assay buffer containing 25 mM Tris/HCl pH 8.5, 25 mM NaCl and 0.1 mM MnCl_2_ at 30 °C. The reaction was started by addition of 35 µM ATP (DisA-Strep) or 10 µM c-di-AMP and pApA (PdeA-Strep), respectively. Samples were taken at indicated time points and the reaction was stopped by incubation at 80 °C for 10 min. To monitor c-di-AMP degradation in cell-free extracts, 100 µL cell-free extract of either *C. glutamicum* wild type or Δ*pdeA* was mixed with 100 µL assay buffer and a final concentration of 10 µM c-di-AMP was added. The reaction mixture was incubated and further treated, as described above.

### 2.5. Detection of Adenylpurines by HPLC

Analysis of adenylpurines was performed based on Katayama et al. [58]. Cell-free extracts of *C. glutamicum* or *E. coli* were prepared by glass bead disruption using a Precellys 24 tissue homogenizer (Bertin Technologies, Montigny-le-Bretonneux, France). For the analysis of cell free extracts, two sample volumes of cold acetonitrile were added, incubated on ice for 5 min and the precipitate was removed by 5 min centrifugation at 15,000× *g*. Derivatization was carried out by mixing 1 sample volume with an equal volume of 1 M Na-acetate pH 4.5 and 0.1 sample volume 50% (*w*/*v*) chloroacetaldehyde solution. The samples were incubated at 80 °C for 20 min, cooled on ice and subsequently analyzed via HPLC using a LaChrom Elite system (Hitachi, Chiyoda, Japan), consisting of a L-2130 gradient pump module, L-2300 column oven and a L-2485 fluorescence detector, as well as an MPS3C autosampler (Gerstel GmbH & Co. KG, Mülheim an der Ruhr, Germany). For separation of nucleotides, 5 µL samples were injected into either a NucleoDur 100-5 C-18 column or a NucleoDur HILIC 5 µM column (Macherey-Nagel GmbH & Co. KG, Düren, Germany). Running conditions for the ion pairing reversed phase (IPC) C-18 column were as follows: flow rate 0.5 mL min^−1^ at 40 °C. Elution started at 90% IPC buffer (30 mM sodium phosphate, 20 mM potassium chloride, 5 mM tetrabutylammonium hydrogen sulfate) and 10% acetonitrile for three min. Acetonitrile concentration was then linearly increased to 15% over 22 min followed by a 3 min isocratic step at 60% acetonitrile and subsequent 10 min re-equilibration of the column at 10% acetonitrile. Hydrophilic interaction chromatography (HILIC) was carried out at a flow rate of 0.8 mL min^−1^ and 35 °C. The separation method started at a concentration of 30% 200 mM ammonium acetate buffer (pH 5.3) and 70% acetonitrile. Ammonium acetate buffer percentage was increased linearly to 42.2% over 8 min, kept constant for 2.5 min and increased linearly to 70% over 1.5 min followed by re-equilibration at 30:70 for 8 min. Detection of fluorescent N6-etheno-purines occurred with excitation and emission wavelengths of 278 and 415 nm, respectively.

### 2.6. Real-Time Quantitative PCR

Relative transcript levels were determined via qPCR [59,60]. For RNA isolation, cell-free extracts of *C. glutamicum* were prepared by glass bead disruption using a Precellys 24 tissue homogenizer (Bertin Technologies, Montigny-le-Bretonneux, France). RNA was isolated using the NucleoSpin^®^ RNA kit (Macherey-Nagel GmbH & Co. KG, Düren, Germany). DNA contaminations in RNA samples were removed by twofold treatment with DNase using the TURBO DNA-free^TM^ kit (Thermo Fisher Scientific, Waltham, MA, USA). RNA was reversely transcribed into complementary DNA (cDNA) using the iScript^TM^ cDNA Synthesis Kit (Bio-Rad Laboratories GmbH, Feldkirchen, Germany). The qPCR was carried out using the iTaq Universal SYBR Green Supermix in a CFX96 Touch Real-Time PCR Detection System (both Bio-Rad Laboratories GmbH) with primers listed in Table S1. Relative expression of *mCherry* compared to the resistance gene *cat* expressed from the pXMJ19 backbone was determined according to the 2^−∆∆C^_T_ method [61].

### 2.7. Fluorescence Reporter Assays

Fluorescent protein formation in bacterial cells was measured using an infinite^®^ M200 plate reader (Tecan Group AG, Männedorf, Switzerland). Cells were resuspended in 0.9% NaCl solution and the OD_600_ was adjusted to 0.1–0.3. An amount of 200 µL of the resuspended cells were transferred to a black 96 well microtiter plate (Sarstedt AG & Co. KG, Nümbrecht, Germany), and fluorescence was recorded at excitation and emission wavelengths of 570 and 610 nm (mCherry), 450 and 502 nm (FbFP, flavin mononucleotide-based fluorescent protein [62]), or 490 and 531 nm (mVenus), respectively.

## 3. Results

### 3.1. DisA Is a Diadenylate Cyclase in C. glutamicum

**Figure 1:**
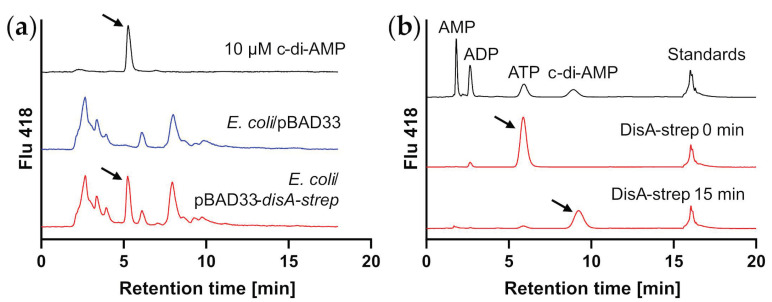
Cyclic diadenosine monophosphate (c-di-AMP) is produced by DisA of *C. glutamicum* ATCC 13032: (**a**) Chromatograms of HILIC-HPLC analysis of 10 µM c-di-AMP cell-free extract of the c-di-AMP-negative organism *E. coli* harboring the plasmid pBAD33 and cell-free extract of *E. coli* harboring the plasmid pBAD33-*disA-strep*. Cells were grown in 2xTY medium containing 1% arabinose; (**b**) IPC-HPLC analysis showing c-di-AMP synthesis by recombinant DisA-Strep from ATP. DisA-Strep was produced in *E. coli*, enriched via affinity chromatography and incubated with 35 µM ATP at 30 °C for 15 min.

**Figure 2:**
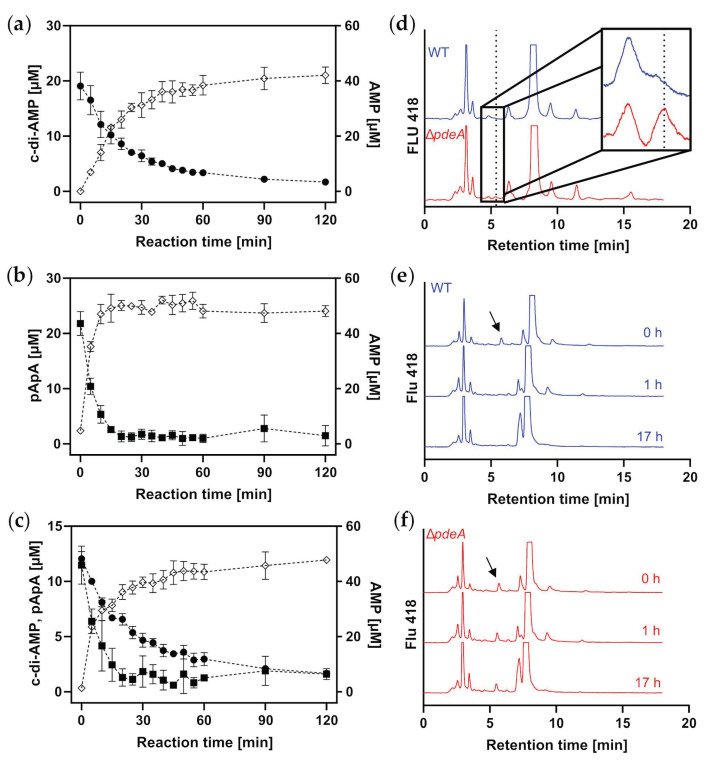
PdeA is the sole c-di-AMP phosphodiesterase in *C. glutamicum*: Recombinant PdeA-Strep was produced in *E. coli* and enriched via affinity chromatography. PdeA-Strep was then incubated at 30 °C with (**a**) cyclic diadenosine monophosphate (c-di-AMP, filled circles), as well as (**b**) phosphoadenylyl-(3′→5′)-adenosine (pApA, filled squares), or (**c**) a combination of both, which are degraded to AMP (open diamonds); (**d**) HILIC-HPLC analysis of cell-free extracts of *C. glutamicum* wild type and Δ*pdeA* with a close-up on the retention time of c-di-AMP slightly above 5 min; (**e**) HILIC-HPLC analysis shows c-di-AMP degradation in cell-free extract of *C. glutamicum* ATCC 13032 but (**f**) not in combination with cell-free extracts of the Δ*pdeA* strain. Cell-free extracts were spiked with c-di-AMP and incubated at 30 °C.

We first sought to identify genes putatively involved in c-di-AMP metabolism in *C. glutamicum*. Whereas homologs to known diadenylate cyclase (DAC) genes, such as *cdaA* or *cdaS*, were not detected, genome analysis revealed a single gene containing the DAC domain, *cg2951*, encoded in an operon together with the DNA-damage-related gene *radA* [63,64,65]. It encodes a 366 amino acid DisA homolog with 41% identity to the well characterized DisA diadenylate cyclase from *B. subtilis*. The gene was thus designated *disA* and was cloned into the *E. coli* expression vector pBAD33, also introducing a C-terminal Strep-tag to the protein. The c-di-AMP-negative [7] host *E. coli* DH5α was transformed with the plasmid pBAD33_*disA-strep*, as well as with the empty vector, and the resulting strains were cultivated in 2xTY medium in presence of the inducer arabinose. The cells were harvested after 8 h; cell-free extracts were prepared and analyzed for adenosine nucleotide derivatives via HPLC. As shown in Figure 1a, a peak corresponding to pure c-di-AMP was observed in the cell-free extract of the strain-expressing *disA-strep*, which was absent in the cell-free extract of the strain carrying the empty vector. In a second approach, DisA-Strep was first produced in *E. coli*, enriched via Strep affinity chromatography from cell-free extract and the protein was then incubated with ATP at 30 °C, and the reaction product was analyzed via HPLC. Figure 1b shows the production of c-di-AMP by the Strep-affinity column enriched DisA-Strep from ATP, where after 15 min the substrate was nearly completely converted to c-di-AMP. We thus concluded that *cg2951/disA* indeed encodes a functional DAC of the DisA-family in *C. glutamicum*. We applied two different established deletion protocols based on homologous recombination and CRISPR [52,66] with selection on complex and minimal medium but were unable to obtain a viable *disA* knockout strain. Although we were not able to detect the c-di-AMP in cell-free extracts of *C. glutamicum* via HPLC (see below, Figure 2d), this suggests that c-di-AMP might be present at rather low concentrations but still be an essential second messenger in *C. glutamicum*, at least under standard conditions. As no other DAC was predicted from the genome sequence, we thus speculate that *disA* encodes the sole and essential DAC in *C. glutamicum*.

### 3.2. PdeA Is the Sole c-di-AMP Phosphodiesterase in C. glutamicum

A genome analysis for putative c-di-AMP phosphodiesterase genes revealed a single gene, *cg2174*, containing the DHH/DHHA1 domains found in c-di-AMP phosphodiesterases [67]. It is encoded as the second gene in an operon of five genes [65]. Its 332 amino acid sequence shares 36 and 20% identity with the homologous proteins from *M. tuberculosis* and *B. burgdorferi*, respectively, and belongs to the group of small, soluble, so-called DhhP-type c-di-AMP PDEs. For initial characterization, a C-terminal Strep-tagged version of the protein was heterologously produced with *E. coli* and enriched via Strep affinity chromatography. The protein was then used for degradation assays, together with the substrates c-di-AMP and its linear degradation intermediate pApA, as well as a combination of both. As shown in Figure 2a–c, PdeA degraded the substrates to about double the amount of the end product AMP. Noticeably, pApA was degraded faster than c-di-AMP, even when both substrates were provided simultaneously. This also remained the case when pApA was provided in a 3:1 surplus over c-di-AMP (Figure S1), suggesting that the intermediate molecule might be immediately further cleaved to AMP and thus might not play a physiological role in *C. glutamicum*. These results show that *cg2174* encodes a functional c-di-AMP/pApA phosphodiesterase, and the gene was thus designated as *pdeA*.

For further characterization, a deletion mutant of *C. glutamicum* lacking the *pdeA* gene was constructed using a CRISPR-Cpf1 method based on Jiang et al. [52]. The strains *C. glutamicum* wild type and the Δ*pdeA* mutant were then grown over night in 50 mL 2xTY medium, and cell-free extracts were prepared and analyzed via HPLC. As shown in the close-up box in Figure 2d, no peak at the retention time of c-di-AMP (dashed vertical line) could be detected in the cell-free extract of the wild type, whereas a small peak was observed for the Δ*pdeA* strain. Cell-free extracts of both strains were then mixed with reaction buffer and spiked with 10 µM c-di-AMP to check whether other PDEs involved in c-di-AMP degradation are existent in *C. glutamicum*. Samples were taken after 0, 1 and 17 h of incubation and analyzed via HPLC. As shown in Figure 2e,f, spiked c-di-AMP was detected in both assays at the reaction start. After 1 h of incubation, c-di-AMP was completely degraded in cell-free extract of the wild type (Figure 2e), whereas it was still fully present in the cell-free extracts of the deletion mutant, even after 17 h (Figure 2f). These results show that only PdeA is responsible for c-di-AMP degradation in *C. glutamicum* under the tested conditions.

### 3.3. C-di-AMP Regulates nlpC via a c-di-AMP Riboswitch

**Figure 3:**
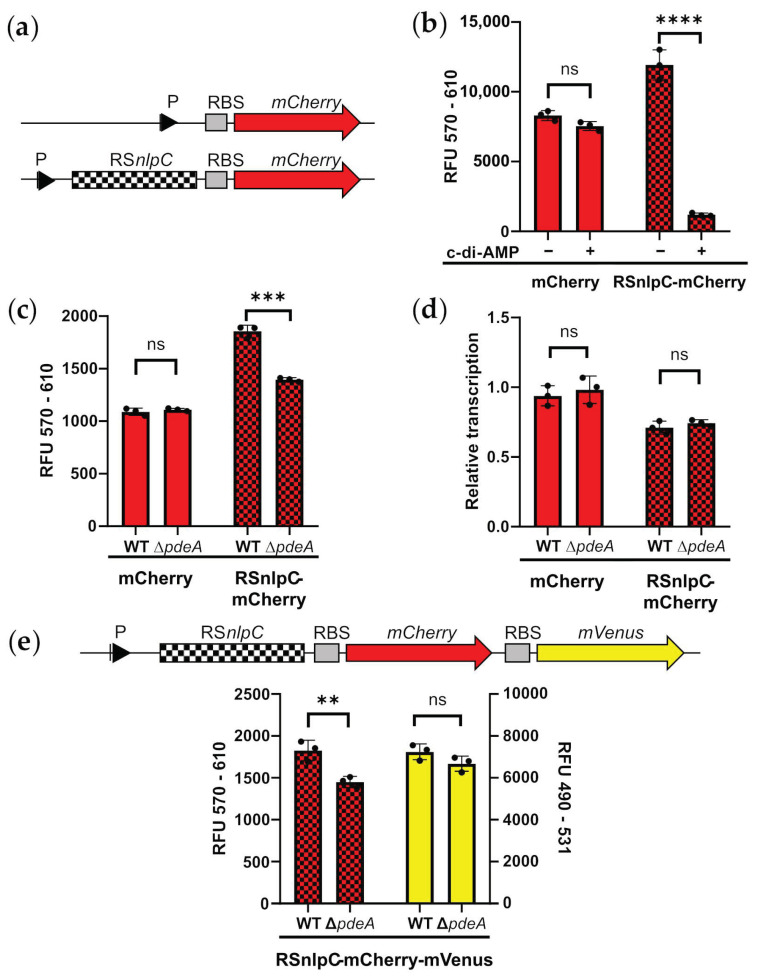
A predicted *ydaO*-type riboswitch (RS*nlpC*) taken from the upstream region of the *nlpC* gene of *C. glutamicum* ATCC 13032 represses reporter expression in presence of c-di-AMP. (**a**) Schematic depiction of reporter constructs, P—promoter (IPTG—inducible), RBS—ribosomal binding site, RS—riboswitch; (**b**) Reporter fluorescence in the cyclic diadenosine monophosphate (c-di-AMP)-negative host *E. coli* co-expressing either a functional or a truncated *disA* gene for presence (+) or absence (−) of c-di-AMP, respectively. Cells were grown at 37 °C in 2xTY medium containing 1% arabinose for induction of *disA/disA’* expression. After 7 h, *mCherry* expression was induced by addition of 1 mM IPTG and relative fluorescence was determined after additional 15 h; (**c**,**d**) Reporter plasmids were introduced into *C. glutamicum* wild type and Δ*pdeA*, and the resulting strains were grown in CGXII minimal medium in presence of 0.1 mM IPTG and relative mCherry fluorescence (**c**), as well as relative *mCherry* transcript levels (**d**), were determined from samples of the mid-exponential growth phase (4 h); (**e**) An additional ribosomal binding site and the reporter gene *mVenus* were cloned downstream of *mCherry* in the riboswitch-reporter plasmid, and the new plasmid was introduced into *C. glutamicum* wild type and Δ*pdeA*. The resulting strains were cultivated, and relative fluorescence levels of both reporter proteins were determined from samples of the mid-exponential growth phase (4 h). N = 3, t—test: ns—not significant, ** *p* < 0.01, *** *p* < 0.001, **** *p* < 0.0001.

One important regulatory feature of c-di-AMP in other bacteria is the control of gene expression via c-di-AMP-sensitive riboswitches. In an in silico analysis, Nelson et al. [26] predicted two putative candidates of these riboswitches in the genome of *C. glutamicum*, one upstream of the resuscitation-promoting factor gene *rpf1* and another upstream of the cell wall peptidase gene *cg2402/nlpC*. As a putative target of c-di-AMP mediated regulation, we selected the riboswitch in front of *nlpC* (RS*nlpC*) for further analysis. The riboswitch sequence was cloned together with the reporter gene *mCherry* under the control of the IPTG-inducible P*_tac_* promoter into the expression vector pXMJ19. A control plasmid with IPTG-inducible *mCherry* expression without riboswitch sequence was also constructed (see Figure 3a). To analyze the influence of the riboswitch in the presence or absence of c-di-AMP in the c-di-AMP-negative host *E. coli*, two plasmids were constructed based on pACYC184 harboring either a full-length or a truncated *disA* gene (*disA’*, obtained by PstI digestion and subsequent religation, resulting in the elimination of 414 bp covering parts of the DAC domain and the linker motif). *E. coli* DH5α was co-transformed with the *mCherry* reporter plasmids together with the *disA* plasmids. The resulting strains thus contained either a functional or disrupted *disA* gene in combination with either a riboswitch-controlled or independent *mCherry* gene. The four strains were grown in 2xTY medium containing 1% arabinose for the induction of *disA/disA’* expression and after 7 h *mCherry* expression was induced by addition of 1 mM IPTG. After another 15 h, mCherry fluorescence was determined. As shown in Figure 3b, mCherry fluorescence was independent on the functionality of the DisA protein for the control plasmid pXMJ19_*mCherry*. In contrast, mCherry fluorescence differed strongly between the strains harboring the functional and disrupted *disA* in case of the reporter plasmid comprising RS*nlpC*. Here, a strong reduction in fluorescence was observed for the strain expressing the functional *disA*. This implies that the combination of functional *disA*- and RS*nlpC*-mediated control leads to reduced gene expression, verifying that RS*nlpC* is indeed a c-di-AMP-responsive ‘OFF’-riboswitch.

To evaluate the functionality of the riboswitch in the native host, we introduced the pXMJ19-based reporter plasmids into *C. glutamicum* wild type and the Δ*pdeA* mutant harboring elevated c-di-AMP levels because of its inability of c-di-AMP degradation (compare Figure 2d–f). As shown in Figure 3c, without riboswitch, reporter fluorescence did not differ between the wild-type-like and the Δ*pdeA* reporter strains, whereas in the presence of RS*nlpC*, fluorescence was lower in the Δ*pdeA* reporter strain compared to the strain with functioning PDE. This, on the one hand, verifies the functionality of the riboswitch in *C. glutamicum* and, on the other hand, confirms that the Δ*pdeA* strain indeed harbors elevated c-di-AMP levels. Furthermore, mutations, as described earlier for the *ydaO* riboswitch [26], led to the loss of c-di-AMP responsiveness of RS*nlpC* (Figure S2). To get insights into the mode of action of the riboswitch, we isolated total RNA from the reporter strains and determined relative *mCherry* transcript levels via RT-qPCR. As shown in Figure 3d, the transcript levels did not differ between the wild-type-like and mutant reporter strains, indicating that the regulation by RS*nlpC* most likely takes place at the level of translation. To further evaluate this finding, we introduced a second reporter gene, *mVenus*, together with a ribosomal binding site (RBS) downstream of the *mCherry* in the riboswitch-containing reporter plasmid (Figure 3e). The plasmid was again introduced into the *C. glutamicum* wild type, and the Δ*pdeA* and fluorescence of both reporter proteins was determined. Whereas mCherry fluorescence was again lower in the Δ*pdeA* reporter strain due to the presence of the riboswitch, mVenus fluorescence did not significantly differ between both strains. This further supports the hypothesis that only the translation of mCherry is affected by the elevated c-di-AMP in Δ*pdeA*. Thus, we conclude, that in contrast to the *ydaO/kimA* riboswitch of *B. subtilis*, the *C. glutamicum* RS*nlpC* most likely acts on a translational level, which has not been shown for c-di-AMP riboswitches before.

### 3.4. The Gene nlpC Is Strongly Regulated but Neglectable for Maintenance of Cell Morphology

**Figure 4:**
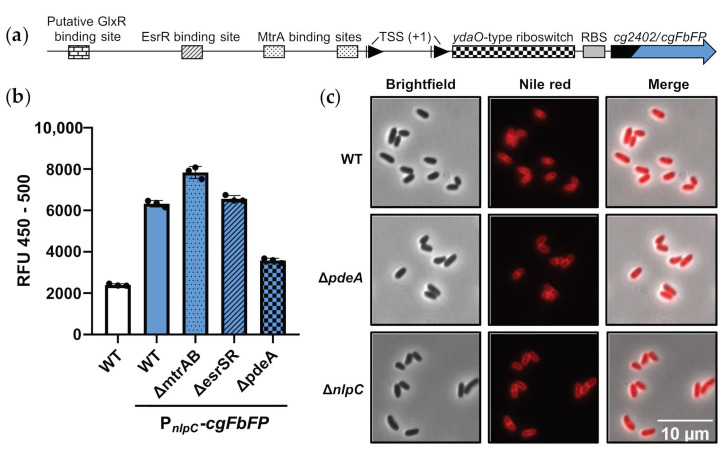
The gene *nlpC* encodes a strongly regulated, non-essential putative cell wall peptidase. (**a**) Schematic depiction of the reporter plasmid comprising the *nlpC* 5′-UTR and the reporter gene *cgFbFP* fused to the first 30 bp of *cg2404/nlpC*; (**b**) The promoter reporter plasmid was introduced into different *C. glutamicum* strains. Strains were grown in CGXII minimal medium and relative reporter fluorescence was determined after 6 h during exponential growth; the wild type without plasmid served as background control. N = 3, shown as mean and standard deviation; (**c**) Fluorescence microscopy pictures of nile-red-stained *C. glutamicum* WT, Δ*pdeA* and Δ*nlpC*.

As stated, the riboswitch-controlled *nlpC* encodes for one of four putative NlpC/P60-type cell wall peptidases in *C. glutamicum*, and the whole operon seems to be strictly regulated by several regulatory systems, i.e., MtrAB, GlxR and EsrISR [39,41,42]. This multi-level regulation suggests an important role of the *nlpC*-operon for *C. glutamicum*. To investigate the effects of the different regulatory elements, a reporter plasmid was constructed as follows. As schematically depicted in Figure 4a, it comprises the *nlpC* promoter region, including the first 30 bp of the *nlpC* gene fused to a *C. glutamicum* codon-optimized version of the reporter gene *ecFbFP*, designated as *cgFbFP*. This gene codes for a flavin-mononucleotide-based fluorescent protein [62], the codon-optimized sequence of *cgFbPF* is listed in Table S2. The reporter plasmid was introduced into the *C. glutamicum* wild type, as well as the mutant strains Δ*mtrAB* [47], Δ*esrSR* [48] (designated as cgtSR7) and Δ*pdeA*. The strains were then grown alongside a wild-type control strain in CGXII medium with 1% glucose and fluorescence was measured in the mid-exponential growth phase. Relative fluorescence for all strains is depicted in Figure 4b. Background fluorescence in the wild-type control was slightly above 2000 RFU, whereas the strain carrying the promoter reporter exhibited more than 6000 RFU. This result proved that the 10 amino-acid-NlpC’-FbFP fusion protein can be used as fluorescence reporter in *C. glutamicum* (most likely also under oxygen-independent conditions). In comparison to the wild-type reporter strain, the Δ*mtrAB* reporter strain showed slightly increased fluorescence of 8000 RFU, indicating that the absence of the repressor MtrA led to a somewhat higher reporter gene transcription. In contrast, the absence of EsrR had no effect on reporter expression. Both results are in accordance with literature reports, where it has been shown that MtrAB is partially activated during growth in the CGXII minimal medium, probably due to its relatively high osmolarity [47]. On the other hand, EsrISR is only active during cell wall stress, e.g., induced by bacitracin treatment [42]. Furthermore, a rather strong repression of the reporter in the Δ*pdeA* reporter strain due to its elevated c-di-AMP level was observed. Altogether, the experiments confirm the multi-level regulation of the *nlpC-*operon, whereas the results in Figure 3 suggest that the riboswitch here also only affects the first gene, *nlpC*, due to its assumed translational regulation mechanism.

As the deletion of DhhP-type PDEs, as well as *nlpC* genes, have been reported to also affect cell morphology [21,40,68], this was also considered for *C. glutamicum*. To further analyze the relevance of NlpC for cell morphology, a Δ*nlpC* mutant was constructed, and this strain, as well as *C. glutamicum* wild type and Δ*pdeA*, were analyzed via fluorescence microscopy. For the better discrimination of single cells and division septa, cell membranes were stained with nile red. Representative pictures of the three bacterial strains are shown in Figure 4c. As can be seen, neither the presumably low translation rate of *nlpC* in the Δ*pdeA* mutant nor the complete absence of *nlpC* led to visible morphological changes when compared to the wild type. Thus, the *nlpC* gene itself, although it represents the leading gene of a strictly regulated operon, seems to be dispensable for *C. glutamicum* at least under the given laboratory conditions.

## 4. Discussion

In our study, we identified and partly characterized the DAC DisA and the DhhP-type PDE PdeA of *C. glutamicum* as enzymes that are involved in c-di-AMP metabolism. In particular, we verified c-di-AMP synthesis from ATP by DisA and showed degradation of c-di-AMP and its linear derivate pApA by PdeA. Several arguments support our hypothesis that those two enzymes are solely responsible for c-di-AMP homeostasis in *C. glutamicum*. As all hitherto characterized DACs contain the DisA/DAC domain [16], the presence of other DACs besides DisA is highly unlikely in *C. glutamicum*. Furthermore, we were not able to delete *disA* using established procedures, indicating a putative essential role for the cell, at least under the tested conditions. It is thinkable that this apparent essentiality could be circumvented, as described for *B. subtilis*. Here, a c-di-AMP-free strain lacking all three DAC-encoding genes could only be obtained at low potassium concentrations as control of this ion is one important role of c-di-AMP in this organism [4]. However, this approach still has to be evaluated for *C. glutamicum*.

Although we were not able to detect c-di-AMP in the *C. glutamicum* wild-type strain, some c-di-AMP accumulation was observed for the Δ*pdeA* mutant, verifying DAC activity of DisA in vivo. Regarding PDEs, we showed that the *pdeA* deletion mutant is not capable of c-di-AMP degradation anymore. Thus, although PdeA belongs to the class of DhhP-type PDEs, which have been described to be mainly responsible for pApA degradation, a two-step mechanism with two different enzymes for c-di-AMP degradation [69] is probably unlikely for *C. glutamicum*. In *S. aureus*, c-di-AMP is first linearized to pApA by the membrane-bound PDE GdpP, which is not capable of further pApA cleavage but is then hydrolyzed to AMP by the DhhP-type Pde2 [69]. The authors further suggested that pApA is additionally actively involved in feedback inhibition of the c-di-AMP hydrolyzing enzyme GdpP. However, recombinantly produced *C. glutamicum* PdeA-Strep degraded both pApA and c-di-AMP, whereas pApA was cleaved faster in all conditions tested, similar to Pde2 of *S. aureus* or *Streptococcus pneumoniae* or TmPDE from *T. maritima* [23,69]. This suggests that the linear intermediate might always be immediately cleaved to AMP in *C. glutamicum*, which would imply that pApA does not fulfill a regulatory function in this organism. Thus, we conclude that c-di-AMP metabolism is most likely mediated solely by DisA and PdeA in *C. glutamicum*. However, it remains to be elucidated how c-di-AMP pools are controlled and adjusted. Additionally, nothing is known about the regulation of *disA* and *pdeA* expression; in addition, *disA* was reported to be located in an operon together with the SOS-response-related gene *radA* [65]. However, an in-depth analysis of the SOS response in *C. glutamicum* only revealed *radA* to be affected by the LexA regulator [70].

As one of the possible targets of c-di-AMP-mediated regulation in *C. glutamicum*, we characterized a putative *ydaO*-type riboswitch located upstream of the cell wall peptidase gene *cg2402*/*nlpC*. Whereas the prototype c-di-AMP riboswitch from *B. subtilis* has been shown to act via premature transcription termination [26], a c-di-AMP riboswitch in *Streptomyces coelicolor* has been reported to lack a canonical terminator structure downstream of the riboswitch and that the regulation is also Rho-independent [28]. Our reporter experiments with the *C. glutamicum* riboswitch RS*nlpC* (which also lacks a canonical terminator sequence) indicated that the presence of c-di-AMP represses the synthesis of reporter fluorophores most likely on a translational level. In particular, in the RS*nlpC* reporter strains, c-di-AMP did not affect reporter transcript levels and only affected the reporter fluorescence of the first gene of an artificial operon. However, St-Onge et al. [28] found that a *Streptomyces* strain lacking the riboswitch sequence exhibited increased transcript levels of the regulated gene. In our study, we observed a similar effect for the reporter transcript levels when comparing the construct with riboswitch against the one without at equal c-di-AMP levels (Figure 3d). It became clear that presence of the riboswitch sequence led to a decrease in transcript levels regardless of the c-di-AMP level, suggesting a c-di-AMP-independent transcription attenuation just by the presence of the riboswitch. In an additional study, St-Onge and Elliot [71] reported the observation of terminated transcripts in the presence of c-di-AMP for the *S. coelicolor* riboswitch in vitro using an *E. coli* RNA polymerase. They further hypothesized that in presence of the *S. coelicolor* RNA polymerase, this effect might be even more pronounced. Our results, in contrast, suggest that for *C. glutamicum* at least, transcription might not be affected by c-di-AMP, at least under the tested conditions, and that RS*nlpC* likely acts on translation. On the molecular level, it is thinkable that the CTCC sequence contained in RS*nlpC* might be responsible for this process. This sequence is complementary to the GGAG region present in both the native ribosomal binding site (RBS) of *nlpC*, as well as the RBS in front of *mCherry* on the reporter plasmid. In theory, the absence of c-di-AMP would lead to the formation of a stem loop, where the CTCC pairs with a GGAG present roughly 20 bases upstream. When c-di-AMP would bind to the riboswitch, this theoretical stem could be relaxed and the exposed CTCC sequence then could block translation by formation of a pseudoknot by pairing with the GGAG present in the RBS. Future investigations to reveal the exact mechanism should involve RNA biochemistry experiments, as well as the crystallization studies of the riboswitch in the presence and absence of c-di-AMP, as is already shown for the transcriptional c-di-AMP riboswitches of Firmicutes [26,72,73].

The *C. glutamicum* gene regulated by the riboswitch, *cg2402/nlpC*, is part of an operon of seven genes, which comprises a second *nlpC*-type gene, and other genes encoding for a glycosyltransferase, a glucokinase, two acyltransferases and a small putative protein [65]. In addition to the two *nlpC* genes in this operon, *C. glutamicum* possesses two other genes belonging to the class of NlpC/P60 proteins. These enzymes are involved in peptidoglycan hydrolysis for cell wall remodeling [38]. However, Tsuge et al. [40] could show that deletion of the *cg2402/nlpC* homolog in *C. glutamicum* R, cgR_2070, does not affect cell morphology, which is in line with our findings for the Δ*nlpC* mutant. In contrast, a defect in cell separation was observed in a mutant lacking another NlpC-like peptidase-encoding gene, cgR_1596 (also described for *M. smegmatis* [74], and *C. glutamicum* MB001 [75]). Additionally, when cgR_2070 was deleted in this strain, severe defects were observed [40]. It is, therefore, conceivable that in the *C. glutamicum* ATCC 13032 Δ*nlpC* mutant, NlpC function would be compensated by the cgR_1596 homolog encoded by *cg1735*, which also suggests a redundant role for *nlpC*. However, the whole *nlpC-*operon appears to be strongly controlled by several mechanisms. Its promoter region comprises binding sites for the master regulator GlxR, as well as the response regulators MtrA and EsrR [39,41,42,65] and the c-di-AMP riboswitch. GlxR is a cyclic AMP-dependent master regulator in *C. glutamicum*, which likely controls 14% of its annotated genes [76,77]. The two-component system MtrAB has been shown to repress *nlpC* and other cell-wall-related genes while activating the expression of genes encoding transporters for compatible solutes under hyperosmotic conditions [39,47,78]. Consequently, the three component system EsrISR also represses *nlpC* but during cell envelope stress, e.g., bacitracin treatment [42]. Whereas these transcriptional regulators most likely affect the whole operon, the supposed translational regulation via RS*nlpC* indicates a modulatory fine-tuning role of c-di-AMP with regard to cell envelope biogenesis in *C. glutamicum*.

In conclusion, we were able to identify c-di-AMP as a second messenger in *C. glutamicum*. The synthesis of the molecule is achieved by the diadenylate cyclase DisA under the consumption of two molecules’ ATP while degradation to AMP is catalyzed by the soluble DhhP-type hydrolase PdeA. Furthermore, we could show that c-di-AMP is involved in the regulation of cell envelope homeostasis by interacting with a presumed translational riboswitch in front of the cell wall hydrolase gene *nlpC*.

## Figures and Tables

**Table 1:** Strains and plasmids used in this study.

Strain or Plasmid	Relevant Characteristics	Source or Reference
Strains		
*Escherichia coli* DH5α	F^-^*thi-1 endA1 hsdR17* (r^–^ m^-^) *sup*E44 Δ*lac*U169 (φ80*lacZ*Δ*M15*) *recA1 gyrA*96 r*elA*1 F^-^ λ^-^*ilvG rfb*-50 *rph*-1	[45]
*Escherichia coli* BL21	*ompT*, *hsdSB* (rB^-^, mB^-^), *dcm*, *gal* (DE3)	[46]
*Corynebacterium glutamicum* ATCC 13032	Wild type	American Type Culture Collection
*C. glutamicum* Δ*pdeA*	ATCC 13032 with in-frame deletion of the cyclic-diadenosine monophosphate (c-di-AMP) phosphodiesterase (PDE) gene *pdeA* (*cg2174*)	This work
*C. glutamicum* Δ*nlpC*	ATCC 13032 with in-frame deletion of the cell wall peptidase gene *nlpC* (*cg2402*)	This work
*C. glutamicum* Δ*mtrAB*	ATCC 13032 with in-frame deletion of the two-component system (TCS) *mtrAB*	[47]
*C. glutamicum* Δ*esrSR*	ATCC 13032 with in-frame deletion of the TCS *esrSR* (*cgtSR7*)	[48]
**Plasmids**		
pACYC184	Cm^r^, Tc^r^, Ori_p15A_	[49]
pACYC_*disA-strep*	Tc^r^, replacement of Cm^r^ region with *araC*-P_BAD_-disA-strep region from pBAD33_*disA-strep*	This work
pACYC_*disA’-strep*	As above, in-frame deletion of 414 bp in *disA* by PstI digestion and subsequent religation	This work
pBAD33	Cm^r^, Ori_p15A_, *araC*, P_BAD_	[50]
pBAD33_*disA-strep*	Expression plasmid carrying the *cg2951/disA* gene from *C. glutamicum* with a C-terminal strep tag	This work
pJC1	Shuttle vector, Km^r^, Ori_p15A_, Ori_pHM1519_	[51]
pJC1_P*_cg2402_*-*cgFbFP*	Promoter reporter comprising 594 bp upstream of the *cg2402/nlpC* gene and including its first 30 bp fused to the reporter gene *cgFbFP*	This work
pJYS3	*cpf1*, Km^r^, Ori_pBL1_, Ori_pSC101_	[52]
pJYS3_d*pdeA*	CRISPR/Cpf1-mediated deletion of of the c-di-AMP PDE gene *pdeA* (*cg2174*)	This work
pJYS3_d*nlpC*	CRISPR/Cpf1-mediated deletion of of the cell wall peptidase gene *nlpC* (*cg2402*)	This work
pXMJ19	Shuttle expression vector, P*_tac_*, *lacI^q^*, Cm^r^, Ori_pMB1_, Ori_pBL1_	[53]
pXMJ19_*mCherry*	Expression plasmid carrying *mCherry* under control of the IPTG inducible P*_tac_* promoter	This work
pXMJ19_RS*nlpC-mCherry*	Expression plasmid carrying *mCherry* under control of the IPTG inducible P*_tac_* promoter and additional control of the riboswitch from the 5′ UTR of *nlpC*	This work
pXMJ19_RS*nlpC-mCherry-mVenus*	As above, with additional control of the putative riboswitch sequence upstream of *cg2402* according to Nelson et al., 2013	This work
pXMJ19_*pdeA-strep*	Cm^r^, IPTG inducible expression of the c-di-AMP PDE gene *pdeA* (*cg2174*)	This work

## Data Availability

All data presented in this study are available in the main body or supplementary files of the manuscript.

## Supplementary Materials

The following supporting information can be downloaded at: https://www.mdpi.com/article/10.3390/microorganisms11020296/s1, Figure S1: PdeA-Strep-mediated degradation of a surplus of pApA in presence of c-di-AMP; Figure S2: Mutations in conserved regions of the riboswitch; Table S1: Oligonucleotides; Table S2: Synthesized Fragment. Supplementary references: [79,80]

## Abbreviations

C-di-AMP 3′,5′-cyclic diadenosine monophosphate; DAC diadenylate cyclase; DhhP c-di-AMP phosphodiesterase from *Borrelia burgdorferi*; PDE phosphodiesterase; pApA 5′-phosphoadenylyl-(3′→5′)-adenosine; IPTG isopropyl β-D-1-thiogalactopyranoside; RBS ribosomal binding site; TCS two-component system.
