# Supplementary material for: C-di-AMP Is a Second Messenger in Corynebacterium glutamicum That Regulates Expression of a Cell Wall-Related Peptidase via a Riboswitch
Source: Microorganisms. 2023 Jan 23;11(2):296. doi: 10.3390/microorganisms11020296 (PMC9960051; doi:10.3390/microorganisms11020296)
Supplement: Supplementary file 1 [file microorganisms-11-00296-s001.zip › microorganisms-2141523-supplementary.pdf]

microorganisms

MDPI

Supplementary Materials

1. Supplementary Methods—Construction of Plasmids

pBAD33_*disA-strep*: The gene *cg2951/disA* was amplified from genomic DNA of *C. glutamicum* using primers disA_fw and disA-strep_rv, introducing a C-terminal Strep-tag. The resulting fragment was ligated to pJET1.2 and verified via sequencing. Then, *disA-strep* was cut from pJET with SacI and KpnI and ligated to pBAD33 digested with the same enzymes, yielding pBAD33_*disA-strep*.

pACYC_*disA-strep* and pACYC_*disA′-strep*: First, a fragment containing the chloramphenicol resistance gene was removed from pACYC184 by restriction digestion with BsaAI. A fragment containing *araC*, P_{BAD} and *disA-strep* was obtained from pBAD33_*disA-strep* by restriction digestion with NaeI and SmaI. This fragment was then ligated to the fragment of pACYC184 containing the tetracycline resistance gene as well as the origin of replication, yielding pACYC_*disA-strep*. The truncation of *disA* was carried out by restriction digestion of pACYC_*disA-strep* with PstI, which cuts twice in *disA*, and subsequent religation. This resulted in an in-frame deletion of 414 bp (138 AA, 38% of the gene/protein), removing parts of the DAC domain as well as the DisA-linker motif.

pJC1_P_{cg2402}-*cgFbFP*: To construct a promoter reporter plasmid for monitoring expression of *cg2402/nlpC*, first, 594 bp upstream of the *nlpC* start codon plus 30 bp of the gene itself were amplified from genomic DNA of *C. glutamicum* using primers Pcg2402_fw and Pcg2402_rv. The *cgFbFP* fragment obtained from Eurofins genomics (sequence: Table S2) was cloned into pJET1.2 and subsequently amplified without start codon using primers *cgFbFP′*_fw and *cgFbFP*_rv. The target plasmid pJC1 was linearized by restriction digestion with BamHI. All three fragments were then assembled in an isothermal reaction to yield pJC1_P_{cg2402}-*cgFbFP*.

pJYS3_d*pdeA* and pJYS3_d*nlpC*: The plasmids for genomic deletion of *cg2174/pdeA* and *cg2402/nlpC* were constructed based on Jiang *et al*. [52] as follows. Respective sgRNA fragments were obtained from overlap extension PCR using primer OE_sg-universal_fw together with the respective rv primers (table S1). Upstream and downstream regions were amplified using the respective primers from genomic DNA of *C. glutamicum*. The three fragments sgRNA, up-region and do-region were then introduced into SmaI/SwaI-digested pJYS3-KH [80] in a single isothermal assembly reaction.

pXMJ19_*mCherry*, pXMJ19_RS*nlpC-mCherry* and pXMJ19_RS*nlpC-mCherry-mVenus*: The reporter gene *mCherry* was amplified from pOGOduet_*mCherry* [81] using primers RBS_*mCherry*_fw and *mCherry*_rv. The resulting fragment was subcloned into pJET1.2 and sequence as well as introduction in forward orientation were verified via sequencing. The plasmid pJET_*mCherry* was digested with XhoI and XbaI and ligated to a SalI and XbaI-linearized pXMJ19 to yield pXMJ19_*mCherry*. The putative riboswitch sequence upstream of *cg2402/nlpC* was amplified from genomic DNA of *C. glutamicum* using primers RS*nlpC*_fw and RS*nlpC*_rv. The resulting fragment was sub-cloned into pJET1.2 and verified via sequencing. The reporter gene *mCherry* was digested from pJET_*mCherry* using BamHI and SalI and ligated to pJET_RS*nlpC* linearized beforehand with the same enzymes. The resulting plasmid contained the fragment RS*nlpC-mCherry*, which was then obtained by restriction digestion with XbaI and EcoRI and ligated to pXMJ19 linearized beforehand with the same enzymes to yield pXMJ19_RS*nlpC-mCherry*. For introduction of the second reporter gene *mVenus* together with a ribosomal binding site downstream of *mCherry*, *mVenus* was amplified using primers RBS_*mVenus*_fw and *mVenus*_rv from pOGOduet_*mVenus* [81]. The PCR-fragment as well as pXMJ19_RS*nlpC-mCherry* where then digested with EcoRI and ligated. Correct orientation and functionality of *mVenus* in the resulting plasmid pXMJ19_RS*nlpC-mCherry-mVenus* was verified via mVenus fluorescence at 530 nm, see main article for measurement details.

pXMJ19_*pdeA-strep*: For recombinant expression of the c-di-AMP phosphodiesterase gene *cg2174/pdeA*, a strep affinity tagged variant was constructed as follows. The putative PDE gene *cg2174* was amplified from genomic DNA of *C. glutamicum* with primers pdeA_fw and pdeA-strep_rv. The resulting fragment was cloned into pJET1.2 and verified via sequencing. The *pdeA*-strep fragment was obtained by restriction digestion of pJET_*pdeA-strep* with BamHI and KpnI and subsequently ligated to pXMJ19 linearized with the same enzymes, yielding pXMJ19_*pdeA-strep*.

2. Supplementary Tables

Table S1. Oligonucleotides used in this study[1].

Oligonucleotide	Sequence (5′ → 3′)[1]
	**Expression of *disA***
disA_fw	GCGC**GAGCTC**AGGAGACTATCTATGACACCAACAACCACTCCTGTATCAAAC
disA-strep_rv	GCTA**GGTACC**TTATTTTTCGAACTGCGGGTGGCTCCAAGCGCTACTTAAACGGCCAAGTCCGTCGG
	**Promoter reporter *cg2402***
Pcg2402_fw	GATCAGCGACGCCGCAGGGGCAACTTCGCTCAAATTCC
Pcg2402_rv	GGAAAGACGCGTTTGAATTGTTGCGACG
cgFbFP'_fw	CAATTCAAACGCGTCTTTCCAGAGCTTTG
cgFbFP_rv	GCTGCAGGTCGACTCTAGAGTTATTCGAGCAGTTTCTCGTAC
	**Deletion of *pdeA* and *nlpC***
OE_sg-universal_fw	GGGCTAGATTGACAGCTAGCTCAGTCCTAGGTATAATGGATCCGAATTTCTACTGTTGTAGATCTACAACAGTAGA AATTC
OE_sg-pdeA_rv	CTGAGCCTTTCGTTTTATTTAAATCATCGATTAAGTTGACGGCACAT
pdeA_up_fw	GCTAGCTGTCAATCTAGCCCGAGGAGTGGTCCACCATG
pdeA_up_rv	ACTCCCCCAAAGCAGCATGAAACTGACTATTATC
pdeA_do_fw	TCATGCTGCTTTGGGGGAGTCTTTGCGAAG
pdeA_do_rv	TGTTCACCGGGCCCTCTCCCCGTTGCCAGCCATGATCATG
pdeA_out_fw	TTTACCCCGACCCTGACTT
pdeA_out_rv	ATCGCAGCTAGAACACCC
OE_sg-nlpC_rv	CTGAGCCTTTCGTTTTATTTAAATTGAGGTAAGGCCGGAGCAGTCATCTACAACAGTAGAAATTC
nlpC_up_fw	GCTAGCTGTCAATCTAGCCCAACTAGTGCCGATTTTTC
nlpC_up_rv	GGAATGGCATGTTTGAATTGTTGCGACG
nlpC_do_fw	CAATTCAAACATGCCATTCCACTCTGCAG
nlpC_do_rv	TGTTCACCGGGCCCTCTCCCGGATCCGTGACTGTGCCAC
nlpC_out_fw	GTTCTTCGTCGTGAGCATT
nlpC_out_rv	CTTCTCAGTCTCCGCGTT
	**Reporter plasmids mCherry**
RBS_mCherry_fw	**GGATCC**AAGGAGTTTTCATGGTGAGCAAGGGCGAG
mCherry_rv	GATCC**GTCGAC**TTACTTGTACAGCTCGTCC
RSnlpC_fw	CGTAGATCCTGCAGCGCCAGCATTACAGAAAC
RSnlpC_rv	**GAATTCGTCGACGGATCC**TTCTTCCTGTTTGCTGACC
RBS_mVenus_fw	AGTC**GAATTC**AAGGAGTTTTCATGGTGAGCAAGGGCGAGG
mVenus_rv	AGTC**GAATTC**TTACTTGTACAGCTCGTCCATG
	**Expression of *pdeA***
pdeA_fw	GCATC**GGATCC**CACCAGTGACGGATAATAGTCAG
pdeA-strep_rv	GCTA**GGTACC**TTATTTTTCGAACTGCGGGTGGCTCCAAGCGCTGCTCAAAGTCGTTGTTAGACATCGACAT
**qPCR**	
q_mCherry_fw	CAAGCTGAAGGTGACCAA
q_mCherry_rv	TCAAGTAGTCGGGGATGT
q_cat_fw	GTGTAGAAACTGCCGGAAA
q_cat_rv	GTGAGCTGGTGATATGGGA

[1]restriction sites are indicated in bold, ribosomal binding sites are underlined.

Table S2. Sequence of synthesized fragment.

Fragment	Sequence (5′ → 3′)
cgFbFP for fusion to *nlpC′*	GCGTCTTTCCAGAGCTTTGGGATTCCAGGACAACTGGAAGTGATCAAGAAGGCACTTGACCATGTTCGTG TAGGTGTCGTCATCACCGATCCAGCTCTTGAGGACAATCCCATTGTCTACGTCAATCAGGGTTTCGTGCA GATGACCGGCTATGAGACAGAGGAAATTCTGGGTAAGAATGCTCGGTTTCTCCAGGGCAAACACACTGA TCCTGCCGAAGTTGACAACATTCGCACTGCGTTGCAGAACAAAGAACCGGTTACGGTGCAGATCCAGAA CTACAAGAAGGATGGAACCATGTTCTGGAACGAACTGAACATCGATCCTATGGAGATCGAGGACAAAA CCTACTTCGTTGGCATCCAAAACGACATCACCAAGCAGAAGGAGTACGAGAAACTGCTCGAATAA

3. Supplementary Figures

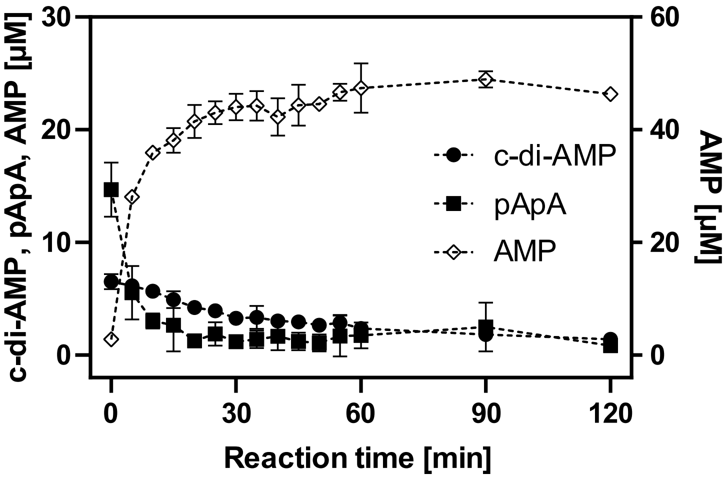

Figure S1. PdeA-Strep-mediated degradation of a surplus of 15 µM phosphoadenylyl-(3′→5′)-adenosine (pApA) in presence of 5 µM cyclic diadenosine monophosphate (c-di-AMP)to AMP. PdeA-strep was produced in *E. coli* and enriched via affinity chromatography. PdeA-strep was then incubated at 30 °C with the substrates. Reaction intermediates were quantified via HILIC-HPLC. Shown are mean and SD of N = 3 replicates.

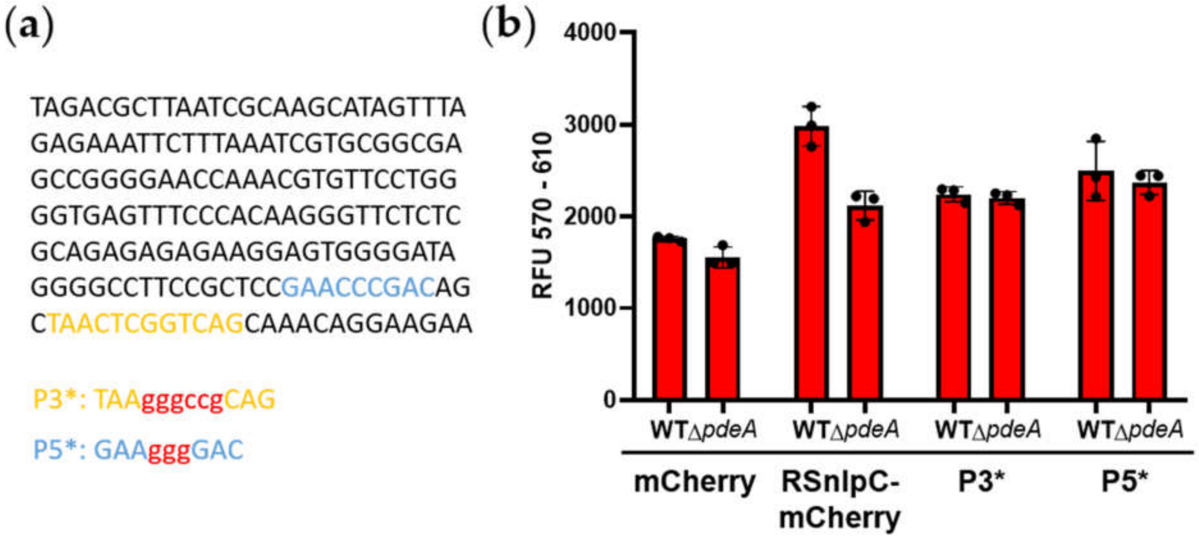

Figure S2. Mutations in conserved regions of the riboswitch based on findings by Nelson *et al*. (2013) [26] were introduced into the *mCherry*-reporter plasmid (compare Fig. 3A). (a) overview of introduced mutations. (b) Relative fluorescence of *C. glutamicum* reporter strains showing loss of c-di-AMP dependency of mutated riboswitches. Shown are mean and SD of N = 3 replicates.
